# Synthesis of a deuterated probe for the confocal Raman microscopy imaging of squalenoyl nanomedicines

**DOI:** 10.3762/bjoc.12.109

**Published:** 2016-06-06

**Authors:** Eric Buchy, Branko Vukosavljevic, Maike Windbergs, Dunja Sobot, Camille Dejean, Simona Mura, Patrick Couvreur, Didier Desmaële

**Affiliations:** 1 Institut Galien (UMR CNRS 8612) Faculté de Pharmacie, Université Paris-Sud, 5, rue Jean-Baptiste Clément, 92296 Châtenay-Malabry, France; 2 Department of Drug Delivery, Helmholtz Centre for Infection Research and Helmholtz Institute for Pharmaceutical Research Saarland, Campus E8.1, 66123 Saarbruecken, Germany; 3 Biopharmaceutics and Pharmaceutical Technology, Saarland University, Campus A 4.1, 66123 Saarbruecken, Germany; 4 BIOCIS (UMR CNRS 8076) Faculté de Pharmacie, Université Paris-Sud, 5, rue Jean-Baptiste Clément, 92296 Châtenay-Malabry, France

**Keywords:** deuterium labelling, nanomedicine, Raman spectroscopy, Shapiro reaction, squalene

## Abstract

The synthesis of ω-di-(trideuteromethyl)-trisnorsqualenic acid has been achieved from natural squalene. The synthesis features the use of a Shapiro reaction of acetone-*d*_6_ trisylhydrazone as a key step to implement the terminal isopropylidene-*d*_6_ moiety. The obtained squalenic acid-*d*_6_ has been coupled to gemcitabine to provide the deuterated analogue of squalenoyl gemcitabine, a powerful anticancer agent endowed with self-assembling properties. The Raman spectra of both deuterated and non-deuterated squalenoyl gemcitabine nanoparticles displayed significant Raman scattering signals. They revealed no differences except from the deuterium peak patterns in the silent spectral region of cells. This paves the way for label-free intracellular trafficking studies of squalenoyl nanomedicines.

## Introduction

Application of nanotechnology to medicine holds promises to profoundly impact healthcare especially to treat severe diseases such as cancer, intracellular infections, neurodegenerative diseases, etc. Indeed, the nanometric size confers to drug delivery systems unique properties which improve the pharmacokinetics and the biodistribution of many active compounds, thus increasing specificity, therapeutic efficacy and reducing systemic exposure and toxicity [1–2]. In recent years, many drug delivery systems have been developed covering all aspects of medicine. Among them, lipid drug conjugates (LDC) were especially developed for the delivery of hydrophilic drugs by covalent coupling with lipid components [3–4]. In this context we recently found that the chemical conjugation of squalene, a natural and biocompatible triterpene, to a drug led to the formation of a prodrug that spontaneously self-assembled as nanoparticles in water. The advantage of this approach is a very high drug loading into the nanoparticles and the absence of burst release [5]. The proof of concept of this method has been done using gemcitabine (**2**), an anticancer chemotherapeutic drug used to treat various solid tumors [6]. Remarkably, the squalene conjugate of gemcitabine (GemSQ) self-assembled in aqueous media as nanoassemblies of around 100 nm mean particle size with a low polydispersity index. The nanosuspension exhibited impressively greater anticancer activity than free gemcitabine against different experimental tumor models [7–11] overcoming the main drawbacks of the parent drug such as its short biological half-life and its low intracellular diffusion [12–13]. Following these initial results, the squalenoylation method was extended to other nucleoside analogues such as antiretroviral agents, ddC, ddI and AZT [14] and to siRNA oligonucleotides [15]. More notably, squalenoylation of adenosine and the subsequent formation of NAs, allowed prolonged circulation of this nucleoside, providing neuroprotection in mice with induced focal cerebral ischemia and in rats undergoing spinal cord injury [16]. Interestingly, the “squalenisation platform” initially developed with highly hydrophilic therapeutics has been further extended to hydrophobic drugs such as beta-lactam antibiotics [17], paclitaxel [18], indolinone kinase inhibitors [19] or doxorubicin [20].

For the elucidation of the mechanisms involved in the efficacy of these promising nanomedicines, the precise knowledge regarding the cellular uptake, the intracellular localization and the determination of the subcellular interactions and trafficking is crucial. To fulfill this task, radioactive labeling or fluorescent probes have been thoroughly used. For example, the subcellular localization of the ^3^H-radiolabeled GemSQ conjugate has been evaluated by micro-autoradiography coupled to confocal imaging of fluorescently labeled cellular structures [21]. A dual radioactive labeling ^3^H,^14^C has been taken into profit to study the pharmacokinetics, the biodistribution and the metabolism of squalenoyl adenosine nanoparticles [22]. Nevertheless, the synthesis of labeled compounds is chemically challenging, expensive and submitted to drastic regulation rules. In addition, the use of fluorescent probes requires the covalent binding of large dye molecules (bodipy, cyanine, rhodamine etc, ...) to the drug conjugate, thus potentially modifying its physicochemical profile as well as the in vivo fate and the pharmacological activity. A simple encapsulation of an amphiphilic fluorochrome in LDC nanoparticles can be used as far as the colloidal stability of the nanocarrier is preserved, but cannot address the intracellular tracking of the loaded drug after carrier disassembling. Thus, specific tools such as fluorescence resonance energy transfer (FRET) and fluorescence quenching, have been developed to study the stability of nanoparticles [23].

**Figure 1:**
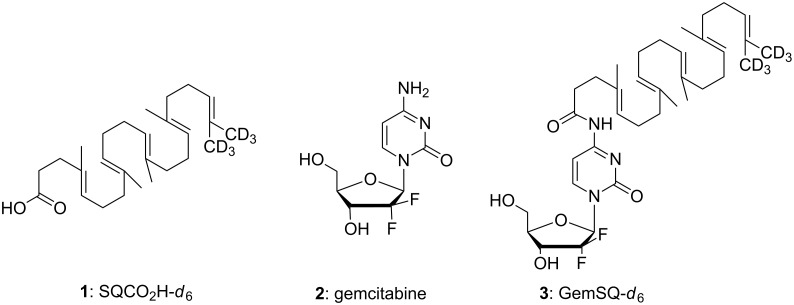
Structure of squalenic acid-*d*_6_, gemcitabine and GemSQ-*d*_6_ conjugate.

In this context, Raman spectroscopy is an interesting technique which is based on the detection of scattered laser light upon irradiating the sample. Nevertheless, because of the low intensity of the Raman scattering, efficient in vivo confocal Raman microspectroscopy of cells had to wait until laser technology and mathematical image processing have made enough progress [24–25]. In contrast to fluorescence spectroscopy, Raman spectroscopy is label-free, as its scattering effect is unique for a specific molecular structure. Raman spectra of cells usually consist of spectral contributions from proteins, lipids and polysaccharides. For the simultaneous detection of both the drug and the cell components with the aim to investigate how they interact, a significant spectral contrast is required. Unfortunately, it can be hardly achieved when dealing with low drug concentrations or biological-like structures such as peptide drugs or nucleoside analogues. To overcome this issue, deuterium can be introduced to a sample molecule, as it exhibits a significant Raman signal at around 2200 cm^−1^, which is in a so called “silent region” (1800–2800 cm^−1^) of most biological molecules. For example as early as 1976, specifically deuterated stearic acids have been used by Sunder et al. in Raman studies and Stiebing et al. studied the uptake of arachidonic acid in human macrophages [26–27]. Furthermore, deuterium does not change the physicochemical properties and thus does not perturb the structure of the described NAs. Consequently, deuterated squalenic acid is expected to be excellent as bioorthogonal Raman tag for squalene-based NAs. Since the Raman signal intensity is expected to increase with the number of deuterium atoms in the same chemical environment, we have undertaken an effort directed towards the synthesis of ω-di-(trideuteromethyl)trisnorsqualenic acid (SQCO_2_H-*d*_6_, **1**) bearing six deuterium atoms on a non-labile position via isotopic exchange. We disclose herein the synthesis of this deuterated Raman probe from natural squalene, its coupling with gemcitabine and the Raman spectra of the deuterated GemSQ nanoassemblies, opening the way to perform intracellular imaging of squalenoyl nanomedicine (Figure 1).

## Results and Discussion

### Chemical synthesis of squalenic acid-*d*_6_ and GemSQ-*d*_6_ conjugate

**Scheme 1:**
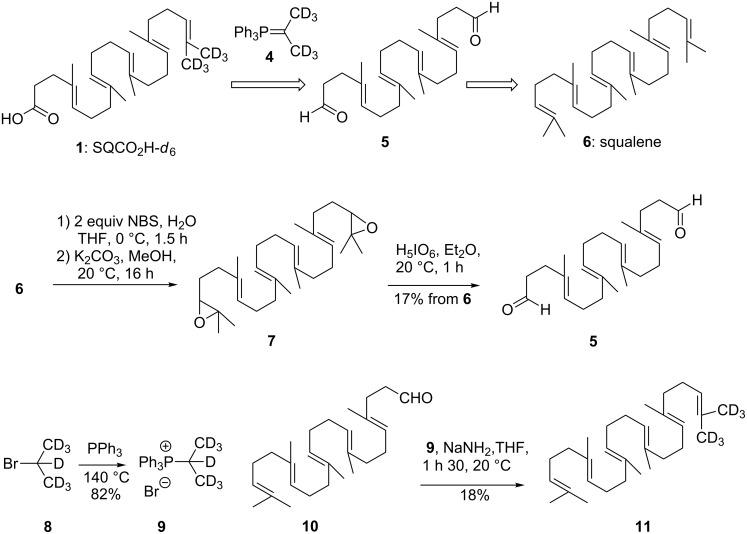
Retrosynthetic route to SQCO_2_H-*d*_6_ (**1**) and synthetic routes for the preparation of dialdehyde **5** and squalene-*d*_6_** 11**.

In a first approach we decided to explore the selective Wittig mono-olefination of dialdehyde **5** readily accessible from the known 2,3;22,23-epoxysqualene (**7**) [28]. As depicted in Scheme 1, the synthetic sequence began with the treatment of squalene with two equivalents of NBS in a water/THF mixture. After separation of the bis-bromohydrin from the monoproduct, potassium carbonate treatment gave the expected diepoxide **7**. Oxidative cleavage with periodic acid provided the corresponding dialdehyde **5** in 17% overall yield from squalene. The perdeuterated phosphonium salt **9** was obtained by simple condensation of commercially available 2-bromopropane-*d*_7_ (**8**) with triphenylphosphine [29]. To our surprise, condensation of dialdehyde **5** with one equivalent of the ylide **4** (**9**, *n*-BuLi, THF, −78 °C) did not afford any amount of the desired deuterated olefin but only polar material that could not be characterized. In an attempt to find more efficient reaction conditions, we investigated this reaction using the simple aldehyde **10** as a model compound, easily accessible from squalene according to the van Tamelen procedure [30]. The condensation of ylide **4** with **10** seemed to be an easy task, but many well-established procedures using various bases (*n*-BuLi, LiHMDS, NaH/DMSO, PhLi) [29,31–32] gave only intractable materials. We finally found that the treatment of **10** with the “instant ylide mixture” of Schlosser made by grinding a solid mixture of **9** and NaNH_2_ [33] delivered the desired squalene-*d*_6_ (**11**) although in a low 18% yield. However, when applied to the dialdehyde **5** this procedure failed to give the expected Wittig adduct.

**Scheme 2:**
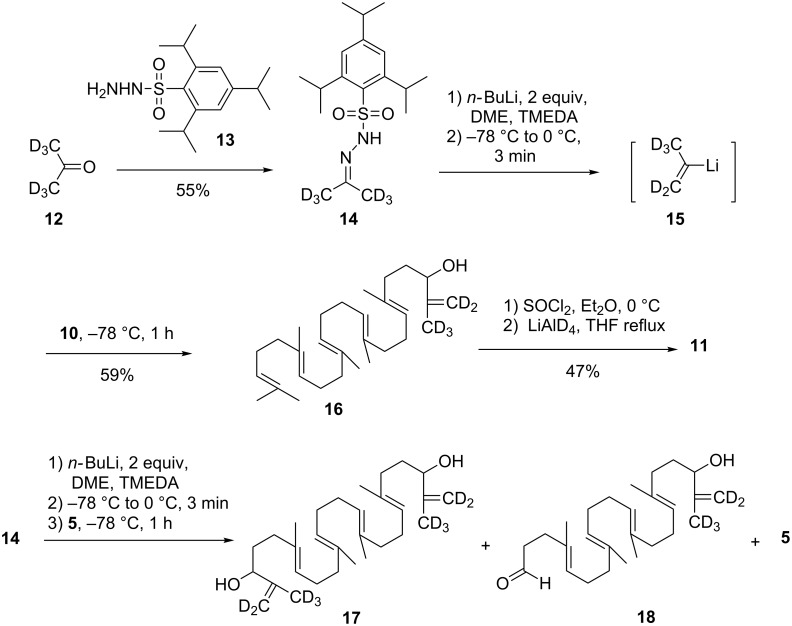
Implementation of the deuterated isopropylidene end-group by the Shapiro reaction of trisylhydrazone of acetone-*d*_6_.

Whatever the origin of this problem, we decided to explore a new strategy based on a more nucleophilic deuterated synthon. In this regard we targeted prop-1-en-2-yllithium-*d*_5_ (**15**) which is easily accessible through the Shapiro reaction of sulfonylhydrazone of acetone-*d*_6_ [34–35]. Thus condensation of trisylhydrazine with acetone-*d*_6_ (99.8% D) gave the expected hydrazone **14** in 55% yield. To our delight, upon treatment with two equivalents of *n-*BuLi and warming to 0 °C, the trisylhydrazone **14** afforded the vinyllithium reagent **15** (along with N_2_ and the trisyl anion) which upon condensation with squalenaldehyde **10** furnished the desired allylic alcohol **16** in 59% yield. Reduction of the hydroxy group of **16** was straightforwardly achieved in 47% yield by treatment with a large excess of thionyl chloride followed by LiAlD_4_ reduction [36]. Having secured an efficient method to reinstall the isopropylidene end-group, we turned our attention to the application of this method to a two-end functionalized squalene derivative. However, when applied to dialdehyde **5** the Shapiro reaction led to a mixture of starting material (30%), allylic alcohol **18** (15%) and diol **17** (20%). This result could not by improved using reverse addition conditions (Scheme 2). Therefore, selective monoprotection of the dialdehyde **5** was next attempted. Unfortunately, treatment of the latter either with ethylene glycol or 2,2-dimethylpropandiol gave a mixture of di- and monoacetal whatever the conditions, as a new example of the lack of chemoselectivity of this long polyisoprenyl chain derivatives.

**Scheme 3:**
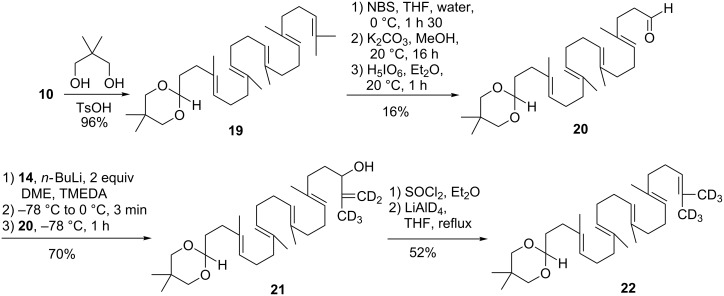
Attempted synthesis of **1** via the protection of the aldehyde **10** as a 5,5-dimethyl-1,3-dioxane.

The differentiation of both ends of squalene was thus performed starting from trisnorsqualenaldehyde **10**. Protection of **10** as 2,2-dimethyl-1,3-dioxane derivative gave **19** in 96% yield which was further elaborated into aldehyde **20** in 16% overall yield according to the three-step van Tamelen sequence (i. NBS, THF, H_2_O; ii. K_2_CO_3_, MeOH; iii. H_3_IO_6_, Et_2_O) [30]. Interestingly enough, the 1,3-dioxane group survived the strongly acidic conditions of the oxidative cleavage. We next turned to the elaboration of the isopropylidene-*d*_6_ moiety. In the event, the Shapiro reaction using trisylhydrazide **14** delivered the expected allylic alcohol **21** in 70% yield. The latter afforded the deuterated ketal **22** in 52% yield, upon sequential treatment with thionyl chloride and LiAlD_4_ as described above. With the success of the implementation of the terminal deuterated isopropylidene group we turned our attention to the deprotection of the ketal and the oxidation of the aldehyde group. This seemingly trivial task, turned out to be unexpectedly challenging. All conditions tried (HCl 3 N, THF, 20 °C; HCl 3 N, THF, reflux; HCl 6 N, dioxane, 20 °C; HCO_2_H, reflux; FeCl_3_·6H_2_O/SiO_2_ [37], Jones reagent) either let the starting material unchanged or induced a complete decomposition. Even treatment with 1,2-ethanedithiol in the presence of BF_3_·OEt_2_ failed to give the corresponding thioketal [38]. These results clearly showed that another protecting group must be used, avoiding the use of an acidic catalyst that triggered the cyclization cascade of the polyisoprenyl chain (Scheme 3).

**Scheme 4:**
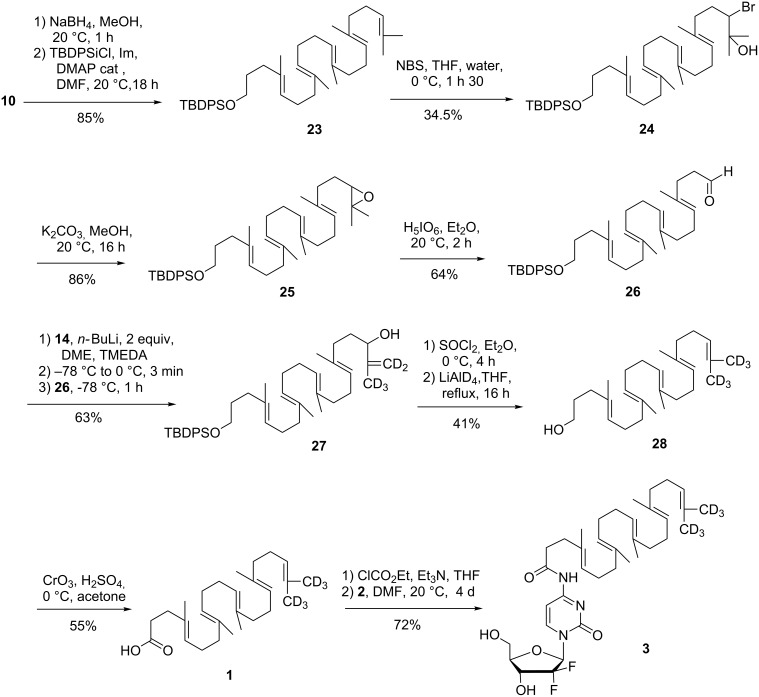
Synthesis of squalenic acid-*d*_6_ **1** and conjugation to gemcitabine.

Despite this setback, the synthetic route seemed suitable to produce the desired material. Thus, the synthetic pathway described above was reimplemented starting from *tert*-butyldiphenylsiloxysqualene **23** readily obtained in 85% yield from squalenaldehyde **10** by NaBH_4_ reduction followed by protection with *tert*-butyldiphenylsilyl chloride. Functionalisation of the opposite extremity of the polyisoprenoid chain using the van Tamelen sequence (i. 1 equiv NBS, THF, H_2_O; ii. K_2_CO_3_, MeOH; iii. H_3_IO_6_, Et_2_O) afforded the aldehyde **26** in 16% overall yield. Uneventfully, the Shapiro reaction with trisylhydrazone **14** produced the allylic alcohol **27**. Thionyl chloride treatment followed by LiAlD_4_ reduction delivered directly the alcohol **28** in 41% yield through concomitant reduction of the intermediate allylic chloride and cleavage of the silyl protecting group. The reductive cleavage of *tert*-butyldiphenylsilyl ethers by LiAlH_4_ has been previously noticed [39]. Jones oxidation straightforwardly completed the synthesis of SQCO_2_H-*d*_6_ **1**. Mass spectral analysis confirmed the presence of the six deuterium atoms (*m*/*z* = 405.3631 for [C_27_H_37_D_6_O_2_^−^]) along a small amount (~5%) of SQCO_2_H-*d*_5_. GemSQ-*d*_6_ **3** was next synthetized using activation with ethyl chloroformate as previously reported [7]. However, to optimize the process a large excess of gemcitabine was used in the reaction to increase the yield to 72% in respect of the more valuable SQCO_2_H-*d*_6_ (Scheme 4).

### Nanoparticle formulation of the GemSQ-*d*_6_ conjugate and Raman spectroscopy

**Figure 2:**
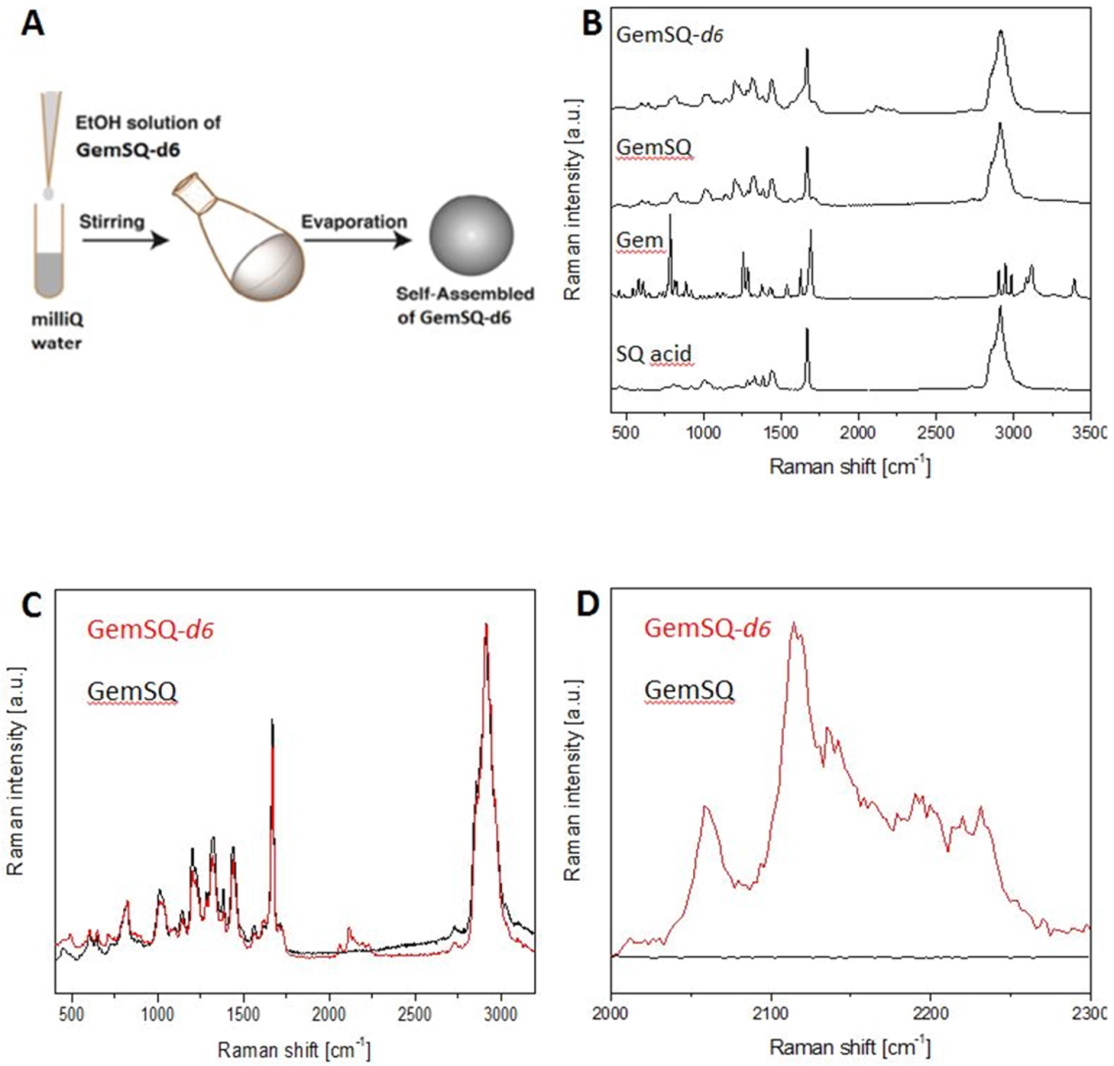
A) Sketch depicting the procedure of preparing the NAs. B) Single Raman spectra of GemSQ-*d*_6_ NAs, GemSQ NAs, gemcitabine and squalenic acid, respectively. C) Single Raman spectra of deuterated GemSQ-*d*_6_ NAs (red) and GemSQ NAs (black). D) Close-up showing the difference between deuterated and non-deuterated compounds.

The GemSQ and GemSQ-*d*_6_ nanoassembly suspensions (2 mg·mL^−1^) were prepared in a single step by nanoprecipitation of an ethanolic solution (2–4 mg·mL^−1^) in milli-Q water [7]. After spontaneous formation of the NAs the organic solvent was evaporated under vacuum (Figure 2A). Single Raman spectra of the raw substances as well as of the particles were recorded (Figure 2B). As depicted in Figure 2C and Figure 2D, the Raman spectra of the deuterated and non-deuterated compounds revealed no differences except the deuterium peaks in the silent region.

## Conclusion

A synthesis of squalenic acid-*d*_6_ was developed through the Shapiro reaction of the sulfonylhydrazone of acetone-*d*_6_ with an ω-silyloxysqualene aldehyde derivative followed by a regioselective reduction of the obtained allylic alcohol. This material was obtained from natural squalene in 0.6% yield over 12 steps. The synthesized deuterated squalenic acid was coupled to gemcitabine to provide the corresponding deuterated squalenoyl conjugate. Raman spectra of the nanoassemblies made of this conjugate were recorded, showing significant Raman peaks in the silent region of the cells thus making this material a potential Raman probe. The use of this material as Raman probe in the study of the intracellular trafficking of GemSQ-based NAs is currently in progress and will be reported in due course.

## Experimental

**(4*E*,8*E*,12*E*,16*E*)-21-(^2^H_3_)methyl-4,8,13,17-tetramethyl(22,22,22-^2^H_3_)docosa-4,8,12,16,20-pentaenoic acid (1):** An ice-cooled solution of alcohol **28** (55 mg, 0.14 mmol) in acetone (2 mL) was treated with a few drops of Jones reagent (CrO_3_/H_2_SO_4_ 6 M) until the mixture took a persistent dark red color. After complete disappearance of the starting material a few drops of isopropanol were added. The mixture was taken into brine (10 mL) and extracted with Et_2_O (4 × 15 mL), dried over MgSO_4_ and concentrated under reduced pressure. The crude product was purified by chromatography over silica gel eluting with petroleum ether/Et_2_O 80:20 to give trisnorsqualenic acid-*d*_6_ (**1**) as a colorless oil (31.5 mg, 55%). ^1^H NMR (CDCl_3_, 300 MHz) δ 5.19–5.07 (m, 5H, =C*H*), 2.45 (t, *J* = 7.6 Hz, 2H, C*H*_2_CO_2_H), 2.30 (t, *J* = 7.6 Hz, 2H, C*H*_2_CH_2_CO_2_H), 2.15–1.95 (m, 16H, =HCC*H*_2_C*H*_2_C(CH_3_)), 1.62 (s, 3H, (C*H*_3_)C=), 1.60 (br s, 9H, (C*H*_3_)C=); ^13^C NMR (CDCl_3_, 75 MHz) δ 179.2 (C, *C*O_2_H), 135.3 (C, CH_2_(CH_3_)*C*=), 135.1 (C, CH_2_(CH_3_)*C*=), 135.0 (C, CH_2_(CH_3_)*C*=), 133.0 (C, CH_2_(CH_3_)*C*=), 131.2 (C, (CD_3_)_2_*C*=), 125.5 (CH, H*C*=), 124.6 (2CH, H*C*=), 124.4 (2CH, H*C*=), 39.9 (2CH_2_), 39.7 (CH_2_), 34.4(CH_2, _*C*H_2_CH_2_CO_2_H), 33.0 (CH_2, _*C*H_2_CO_2_H), 28.4 (2CH_2_), 26.9 (CH_2_), 26.8 (2CH_2_), 16.1 (3CH_3_), 16.0 (CH_3_); IR (film, cm^−1^) ν: 3500–2600 (broad), 2962, 2916, 2856, 2222, 2188, 1709, 1666, 1450, 1411, 1302, 1299, 1259, 1211, 1096, 1049, 955, 846, 736, 704; HRMS–ESI^−^: calcd for C_27_H_37_D_6_O_2_: 405.3645; found: 405.3631.

**(4*E*,8*E*,12*E*,16*E*)-*N*-{1-[(2*R*,5*R*)-3,3-difluoro-4-hydroxy-5-(hydroxymethyl)oxolan-2-yl]-2-oxo-1,2-dihydropyrimidin-4-yl}-21-(^2^H_3_)methyl-4,8,13,17-tetramethyl(22,22,22-^2^H_3_)docosa-4,8,12,16,20-pentaenamide (3)**: To a solution cooled at −5 °C of trisnorsqualenic acid-*d*_6_ (**1**) (31.5 mg, 0.077 mmol) in dry THF (0.6 mL) was sequentially added triethylamine (60 mg, 0.23 mmol) and ethyl chloroformate (10 mg, 0.093 mmol). The reaction mixture was stirred for 30 min at this temperature and a solution of gemcitabine base (60.6 mg, 0.23 mmol) in DMF (2 mL) was added. The reaction mixture was stirred for 4 days at 20 °C and concentrated under reduced pressure. The residue was directly chromatographed over silica gel eluting with cyclohexane/AcOEt 4:1 followed by neat AcOEt to provide GemSQ-*d*_6_ (**3**) as a colorless oil (36.0 mg, 72%). ^1^H NMR (CDCl_3_, 400 MHz) δ 9.15 (br s, 1H, N*H*CO), 8.10 (d, *J* = 7.5 Hz, 1H, H-6), 7.47 (d, *J* = 7.5 Hz, 1H, H-5), 6.18 (t, *J* = 7.4 Hz, 1H, H-1’), 5.20–5.06 (m, 5H, =C*H*), 4.55–4.41 (m, 1H, H-3’), 4.15–3.95 (m, 3H, H-4’, H-5’, O*H*), 3.91 (d, 1H, *J* = 10.8 Hz, H-5’), 2.55 (2H, t, *J* = 7.6 Hz, C*H*_2_CON), 2.32 (2H, t, *J* = 7.4 Hz, C*H*_2_CH_2_CON), 2.10–1.91 (m, 16H, =CC*H*_2_C*H*_2_C(CH_3_)), 1.60 (3H, s, =C(*C*H_3_)), 1.59 (s, 6H, =C(*C*H_3_)), 1.58 (3H, s, =C(*C*H_3_)); ^13^C NMR (CDCl_3_, 75 MHz) δ 173.6 (C, *C*ONH), 163.1 (C, C-4), 155.8 (C, C-2), 145.6 (CH, C-6), 135.3 (C, (CH_3_)*C*= CH_2_(CH_3_)*C*=), 135.0 (2C, CH_2_(CH_3_)*C*=), 132.8 (C, CH_2_(CH_3_)*C*=), 131.2 (C, (CD_3_)_2_*C*=), 126.0 (CH, H*C*=), 124.5 (2CH, H*C*=), 124.4 (2 H*C*=), 122.5 (CF_2_, t, *J* = 258 Hz, C-2’), 97.8 (CH, C-5), 81.8 (CH, C-4’), 69.3 (CH, m, C-3’), 60.0 (CH_2_, C-5’), 39.9 (2CH_2_), 39.7 (CH_2_), 36.7 (CH_2_, NHCO*C*H_2_CH_2_), 34.5 (CH_2_, NHCOCH_2_*C*H_2_), 29.8 (CH_2_), 28.4 (2CH_2_), 27.0 (CH_2_), 26.9 (2CH_2_), 26.8 (2CH_2_), 16.2 (2CH_3_), 16.1 (CH_3_), 16.0 (CH_3_); IR (film, cm^−1^) ν: 3500–3000 (broad), 2979, 2932, 2872, 2852, 2222, 2191, 1724, 1683, 1660, 1618, 1561, 1494, 1433, 1397, 1383, 1365, 1337, 1320, 1312, 1272, 1206, 1194, 1134, 1086, 1069, 1051, 915, 893, 813, 787, 738; HRMS–ESI^−^: calcd for C_36_H_46_D_6_N_3_O_5_F_2_: 650.4257; found: 650.4230.

**Preparation of nanoassemblies from GemSQ and GemSQ-*d*_6_**: In a similar manner to the procedure published [19] the prodrugs based nanoparticle suspensions (2 mg·mL^−1^) were prepared in a single step by dropwise addition of an ethanol solution (4 mg·mL^−1^) in milli-Q water (1 mL) under vigorous stirring (500 rpm). Formation of NAs occurred immediately. After being stirred for 2 min, the nanoparticle suspension was then transferred into a weighted round bottom flask and ethanol was evaporated using a Rotavapor with a preheated water bath (35 °C) setting the vacuum to about 15–50 mbar for about 5 min. Then, the flask was dipped into a water bath (water temperature 37 °C) for about 3–5 minutes. Evaporation was continued till the weight of the contents decreased to 0.8–0.9 g. Then, the volume of the suspension in the flask was made-up to 1.0 g using either 5% dextrose solution or milli-Q water. The colloidal dispersions were stored at 4 °C.

**Raman microscopy measurements.** Confocal Raman microscopy measurements were performed with a WITec alpha 300R+ (WITec GmbH, Ulm, Germany). The excitation source was a diode laser with a wavelength of 532 nm adjusted to a power of 40 mW before the objective. A confocal pinhole of 50 μm rejected signals from out-of-focus regions. An objective with 50× magnification (N.A. 0.8, Epiplan Neofluar, Zeiss, Germany) was applied for acquiring single Raman spectra of the pure compounds with an integration time of 2 s and 10 accumulations. All spectra were background subtracted and normalized to the most intense peak.

## Supporting Information

File 1 Experimental procedures and ^1^H and ^13^C NMR spectral data for compounds **1**, **3**, **11**, **25**, **27**, **28**.
