# Supplementary material for: Synthesis of a deuterated probe for the confocal Raman microscopy imaging of squalenoyl nanomedicines
Source: Beilstein J Org Chem. 2016 Jun 6;12:1127–35. doi: 10.3762/bjoc.12.109 (PMC4979966; doi:10.3762/bjoc.12.109)
Supplement: File 1 — Experimental procedures and 1H and 13C NMR spectral data for compounds 1, 3, 11, 25, 27, 28. [file Beilstein_J_Org_Chem-12-1127-s001.pdf]

Supporting Information

for

Synthesis of a deuterated probe for the confocal Raman microscopy imaging of squalenoyl nanomedicines

Eric Buchy[1], Branko Vukosavljevic[2,3], Maike Windbergs[2,3], Dunja Sobot[1], Camille Dejean[4], Simona Mura[1], Patrick Couvreur[1], and Didier Desmaële*[1]

Address: [1] Institut Galien (UMR CNRS 8612) Faculté de Pharmacie, Université Paris-Sud, 5, rue Jean-Baptiste Clément, 92296 Châtenay-Malabry, France, [2]Department of Drug Delivery, Helmholtz Centre for Infection Research and Helmholtz Institute for Pharmaceutical Research Saarland, Campus E8.1, 66123 Saarbruecken, Germany, [3]Biopharmaceutics and Pharmaceutical Technology, Saarland University, Campus A 4.1, 66123 Saarbruecken, Germany and [4]BIOCIS (UMR CNRS 8076) Faculté de Pharmacie, Université Paris-Sud, 5, rue Jean-Baptiste Clément, 92296 Châtenay-Malabry, France

Email: Didier Desmaële - didier.desmaele@u-psud.fr

*Corresponding author

Experimental procedures and ^{1}H and ^{13}C NMR spectral data for compounds 1, 3, 11, 25, 27, 28

2,4,6-Tris(propan-2-yl)-*N*'-[($^2H_6$)propan-2-ylidene]benzene-1-sulfonohydrazide (14). A mixture of trisylhydrazine (2.5 g, 0.84 mmol) in acetone-d_6 (15 mL) was stirred for 2 h at 20 °C. The reaction mixture was concentrated under reduced pressure and the obtained solid recrystallized in a mixture of MeOH/H_2O, 90:10 (5 mL) to give trisylhydrazone **14** (1.6 g , 55%). Mp. 155 °C (dec); ^{1}H NMR ($CDCl_3$, 300 MHz) δ 7.70-7.20 (br s, 1H, NH), 7.16 (s, 2H), 4.27 (hept, *J* = 6.75 Hz, 2H), 2.90 (hept, *J* = 6.75 Hz, 1H), 1.27 (d, *J* = 6.75 Hz, 12 H), 1.25 (d, *J* = 6.75 Hz, 6 H); ^{13}C NMR ($CDCl_3$, 75 MHz) δ 154.4 (C, *C*=NNH), 153.2 (C, C-4), 151.5 (2C, C-2, C-6), 131.6 (C, C-1), 123.9 (2CH, C-3, C-5), 34.2 (CH), 30.0 (2CH), 29.4 (4CH_3), 23.7 (2CH_3).

(6*E*,10*E*,14*E*,18*E*)-2-($^2H_3$)Methyl-6,10,15,19,23-pentamethyl(1,1-$^2H_2$)tetracosa-1,6,10,14,18,22-hexaen-3-ol (16) A solution of *n*-butyllithium in hexane 0.6 mL, 2.5 M, 1.5 mmol) was added dropwise to a solution of trisylhydrazone **14** (247 mg, 0.71 mmol) in DME (2.5 mL) and TMEDA (0.25 mL, 1.66 mmol) cooled at −78 °C. The reaction mixture was stirred at −78 °C for 30 min. and warmed up to 0 °C. After few min the yellow reaction mixture turned green and a strong gas evolution took place. After completion of the nitrogen evolution, the flask was replaced in the −78 °C cool bath. A solution of aldehyde **10** (250 mg, 0.65 mmol) in DME (0.6 mL) was added dropwise. The mixture was stirred at −78 °C for 1 h and then quenched by a solution of 0.1 N HCl (2 mL). The mixture was taken up into Et_2O (30 mL), the phases were separated and the aqueous layer extracted with Et_2O (4 × 30 mL). The combined organic layers were washed with saturated $NaHCO_3$ (2 mL), brine (2 mL), dried over $MgSO_4$ and concentrated under reduced pressure. The crude product was purified by chromatography over silica gel eluting with petroleum ether/Et_2O 90:10 to provide alcohol **16** as a colorless oil (160 mg, 59%) as a colorless oil. ^{1}H NMR ($CDCl_3$, 300 MHz) δ 5.21-5.08 (m, 4H, =C*H*), 4.04 (dd, *J* = 6.9 Hz, *J* = 5.7 Hz, 1H, C*H*OH), 2.15-1.924 (m, 18 H, =HCCH_2CH_2C(CH_3)), 1.68 (s, 3H, (CH_3)$_2$C=), 1.75-1.60 (m, 17 H); ^{13}C NMR ($CDCl_3$, 75 MHz) δ 147.2 (C, D_2C=*C(*CD_3*)*), 135.0 (C, CH_2(CH_3)*C*=), 134.9 (C, CH_2(CH_3)*C*=), 134.8 (C, CH_2(CH_3)*C*=), 134.6 (C, CH_2(CH_3)*C*=), 131.1 (C, D_2C=*C(*CD_3*)*), 124.7 (CH, H*C*=), 124.4 (2CH, H*C*=), 124.2 (2CH, H*C*=), 75.5 (CH, *C*HOH), 39.6 (3CH_2), 35.6 (CH_2), 33.1 (CH_2), 28.2 (2CH_2), 26.7 (CH_2), 26.6 (2CH_2), 25.6 (CH_3, (*C*H_3)$_2$C=), 17.6 (CH_3), 15.9 (4CH_3); IR (film, cm^{-1}) ν : 3550-3200, 2984, 2965, 2933, 2854, 2843, 2253, 2208, 1667, 1455, 1450, 1383, 1330, 1305, 1151, 1108, 1090, 1044, 1017, 984, 928, 918, 897, 849, 836, 800, 716, 695 ; MS (APCI+) *m/z* (%) 432.2 (40) $[M+H]^+$, 414.2 (100) $[M-H_2O+H]^+$.

ω-Di-(trideuteromethyl)-squalene (11). A mixture of sodium amide (58.5 mg, 1.50 mol) and heptadeutero-isopropyltriphenylphosphonium bromide (**9**, 588 mg, 1.50 mol) was finely crushed in a mortar under inert atmosphere. The solid mixture was transferred to a round-bottom flask and cooled to 0 °C. THF (10 mL) was added dropwise and the white suspension was stirred for 5 min. The mixture was warmed up to 20 °C and stirred for a further 15 min. period. A solution of aldehyde **10** (385 mg, 1.0 mmol) in THF (53 mL) was added dropwise and the resulting mixture was stirred for 1h30. The mixture was cooled to 0 °C and 0.1 N HCl (15 mL) was added. The organic solvent was removed under reduced pressure and the aqueous phase extracted with AcOEt (4 × 20 mL). The combined organic layers were washed with brine (2 mL), dried over $MgSO_4$ and concentrated under reduced pressure. The crude product was purified by chromatography over silica gel eluting with petroleum ether to deliver the title compound as a colorless oil (112 mg, 18% yield). ^{1}H NMR ($CDCl_3$,

300 MHz) δ 5.18-5.11 (m, 6H, =C*H*), 2.15-1.95 (m, 20H, =HCCH_2CH_2C(CH_3)), 1.70 (s, 3H, (CH_3)$_2$C=), 1.64 (s, 15H, =C(CH_3)); ^{13}C NMR ($CDCl_3$, 75 MHz) δ 135.2 (2C, CH_2(CH_3)*C*=), 135.0 (2C, CH_2(CH_3)*C*=), 131.3 (2C, (CH_3)$_2$*C*=), 124.6 (2CH, H*C*=), 124.5 (2CH, H*C*=), 124.4 (2CH, H*C*=), 39.9 (4CH_2), 28.4 (4CH_2), 26.9 (4CH_2), 26.8 (4CH_2), 25.8 (CH_3, (*C*H_3)$_2$C=), 17.8 (CH_3), 16.1 (4CH_3); IR (film, cm^{-1}) ν : 2983, 2928, 2855, 2845, 2226, 2189, 2176, 1667, 1451, 1432, 1383, 1376, 1330, 1223, 1149, 1109, 1049, 984, 889, 849, 835, 724; (ESI+) : Calcd for $C_{30}H_{45}D_6$, 417.4362, found 417.4338.

***tert*-Butyl({[(4E,8*E*,12*E*,16*E*)-4,8,13,17,21-pentamethyldocosa-4,8,12,16,20-pentaen-1-yl]oxy})diphenylsilane (23).** To a solution of 1,1',2-trisnorsqualenol [1] (6.00 g, 15.5 mmol) in dry DMF (22 mL) was sequentially added imidazole (2.10 g, 31.0 mmol), *tert*-butyldiphenyl chlorosilane (4.73 g, 17.2 mmol) and a catalytic amount of DMAP (30 mg, 0.25 mmol). The reaction mixture was stirred at 20 °C for18 h and then the reaction mixture was concentrated under reduced pressure (0.5 mm Hg).The residue was taken up into 0.1 N HCl (15 mL) and the mixture was extracted with AcOEt (4 × 50 mL). The combined organic layers were washed with saturated $NaHCO_3$ (10 mL), brine (10 mL), dried over $MgSO_4$ and concentrated under reduced pressure. The crude product was purified by chromatography over silica gel eluting with petroleum ether and then petroleum ether/Et_2O 95:5. The silyl ether **23** was obtained as a colorless oil (9.3 g, 96%). ^{1}H NMR ($CDCl_3$, 300 MHz) δ 7.80-7.70 (m, 4H, PhSi), 7.45-7.35 (m, 6H, PhSi), 5.18-5.14 (m, 5H, =C*H*), 3.69 (t, *J* = 6.4 Hz, 2H, CH_2OSi), 2.12-2.03 (m, 18 H, =HCCH_2CH_2C(CH_3)), 1.72 (s, 3H, =C(CH_3)$_2$), 1.65 (m, 14H, C$H_2$$CH_2$OSi, =CH(C$H_3$)), 1.61 (s, 3H), 1.10 (s, 9H, (CH_3)$_3$CSi); ^{13}C NMR ($CDCl_3$, 75 MHz) δ 135.7 (4 CH, PhSi), 135.2 (3C, =*C*(CH_3), *C*Si), 135.0 (C, (CH_3)$_2$*C*=), 134.7 (C, =*C*(CH_3)), 134.3 (C, =*C*(CH_3)), 131.3 (C, =*C*(CH_3)), 129.6 (2CH, PhSi), 127.7 (4CH, PhSi), 124.3 (2CH, =*C*H), 124.4 (3CH), 63.6 (CH_2, *C*H_2OSi), 39.9 (2CH_2), 35.9 (CH_2), 31.1 (CH_2), 28.4 (2CH_2), 27.0 (3CH_3, (*C*H_3)$_3$CSi), 26.9 (2CH_2), 26.8 (CH_2), 25.8 (CH_3, (*C*H_3)$_2$C=), 19.3 (C, (CH_3)$_3$*C*Si), 17.8 (CH_3, (*C*H_3)$_2$C=), 16.2 (4CH_3), 16.1 (CH_3) ; IR (film, cm^{-1}) ν : 3072, 2959, 2929, 2856, 1666, 1462, 1447, 1428, 1384, 1377, 1361, 1188, 1112, 1105, 1088, 1029, 1007, 998, 940, 823, 741, 724 ; HRMS (ESI+) : Calcd for $C_{43}H_{64}O_4SiNa$, 647.4618, found 647.4609.

(6*E*,10*E*,14*E*,18*E*)-3-Bromo-22-[(*tert*-butyldiphenylsilyl)oxy]-2,6,10,15,19-pentamethyldocosa-6,10,14,18- tetraen-2-ol (24). To a solution of silyl ether **23** (5.9 g, 9.44 mmol) in a THF (68 mL) water mixture (3.5 mL) cooled at 0 °C, was added by small portions 2.2 g (12.3 mmol) of NBS. The mixture was stirred for 1 h 30 and a saturated aqueous sodium bisulfite solution(10 mL) was added. The reaction mixture was concentrated under reduced pressure and the mixture was extracted with AcOEt (4 × 50 mL). The combined organic layers were washed with saturated $NaHCO_3$ (10 mL), brine (10 mL), dried over $MgSO_4$ and concentrated under reduced pressure. The crude product was purified by chromatography over silica gel eluting with petroleum ether, petroleum ether/Et_2O 95:5 and finally petroleum ether /Et_2O 90:10 to give bromohydrin **24** as a pale yellow oil (2.46 g, 34.5%) along with 1.4 g of the starting silyl ether **23** (29%). ^{1}H NMR ($CDCl_3$, 300 MHz) δ 7.70-7.64 (m, 4H, SiPh), 7.45-7.34 (m, 6H, SiPh), 5.24-5.07 (m, 4H, =C*H*), 3.99 (dd, *J* = 11.4 Hz, *J* = 1.8 Hz, 1H, C*H*Br), 3.64 (t, *J* = 6.4 Hz, 2H, CH_2OSi), 2.40-1.90 (m, 16 H, =HCCH_2CH_2C(CH_3)), 1.85-1.56 (m, 16 H, C$H_2$$CH_2$OSi, =CH(C$H_3$), C$H_2$CHBr), 1.35 (s, 3H, (C$H_3$)$_2$C(OH)), 1.33 (s, 3H, (C$H_3$)$_2$C(OH)), 1.06 (s, 9H, (C$H_3$)$_3$CSi); ^{13}C NMR ($CDCl_3$, 75 MHz) δ 135.7 (4 CH, PhSi), 135.2 (2C, CSi), 135.0 (C, =*C*(CH_3)), 134.7 (C, =*C*(CH_3)), 134.3 (C, =*C*(CH_3)), 133.1 (C, =*C*(CH_3)), 129.6 (2CH, PhSi), 127.7 (4CH, PhSi), 126.2 (CH, =*C*H), 124.6 (CH, =*C*H),

124.5 (CH, =*C*H), 124.4 (CH, =*C*H), 72.6 (C, $(CH_3)_2$*C*OH), 71.1 (CH, *C*HBr), 63.7 (CH_2, *C*H_2OSi), 39.9 (CH_2), 39.8 (CH_2), 38.3 (CH_2), 35.9 (CH_2), 32.3 (CH_2), 31.1 (CH_2), 28.4 ($3CH_2$), 27.0 ($3CH_3$, (*C*$H_3)_3$CSi), 26.9 (CH_2), 25.9 ($2CH_3$, (*C*$H_3)_2$COH), 19.4 (C, $(CH_3)_3$*C*Si), 16.3 (CH_3), 16.2 (CH_3), 16.1 (CH_3), 16.0 (CH_3); IR (film, cm^{-1}) ν : 3600-3300, 3070, 3052, 2960, 2930, 2856, 1665, 1590, 1473, 1462, 1447, 1361, 1333, 1260, 1188, 1112, 1105, 1089, 1029, 1008, 998, 961, 909, 823.

***tert*-Butyl({[(4*E*,8*E*,12*E,*16*E*)-19-(3,3-dimethyloxiran-2-yl)-4,8,13,17-tetramethylnonadeca-4,8,12,16-tetra-en-1-yl]oxy})diphenylsilane (25).** 2.1 g of K_2CO_3 (15.3 mmol) were added to a solution of bromohydrin **24** (3.68 g, 5.1 mmol) in methanol (40 mL). After being stirred for 16 h at 20 °C the reaction mixture was concentrated under reduced pressure. The residue was taken up into 0.5 N HCl (30 mL) and the mixture was extracted with AcOEt (4 × 50 mL). The combined organic layers were washed with saturated $NaHCO_3$ (10 mL), brine (10 mL) and dried over $MgSO_4$. The mixture was concentrated under reduced pressure and the crude product was purified by chromatography over silica gel eluting with petroleum ether and then petroleum ether/Et_2O 98:2 to provide epoxide **25** as a colorless oil (2.75 g, 86%). ^{1}H NMR ($CDCl_3$, 300 MHz) δ 7.71-7.64 (m, 4H, SiPh), 7.45-7.33 (m, 6H, SiPh), 5.24-5.02 (m, 4H, =C*H*), 3.64 (t, *J* = 6.4 Hz, 2H, C*H*$_2$OSi), 2.70 (t, *J* = 6.15 Hz, 1H, $(CH_3)_2$CC*H*(O)), 2.25-1.90 (m, 16 H, =HCC*H*$_2$C*H*$_2$C(CH_3)), 1.72-1.52 (m, 16H, C*H*$_2$CH$_2$OSi, =CH(C*H*$_3$), C*H*$_2$CH(O)), 1.30 (s, 3H, (C*H*$_3)_2$C(O)), 1.26 (s, 3H, (C*H*$_3)_2$C(O)), 1.05 (s, 9H, (C*H*$_3)_3$CSi); ^{13}C NMR ($CDCl_3$, 75 MHz) δ : 135.7 (4 CH, PhSi), 135.2 (2C, CSi), 135.0 (C, =*C*(CH_3)), 134.7 (C, =*C*(CH_3)), 134.3 (C, =*C*(CH_3)), 134.1 (C, =*C*(CH_3)), 129.6 (2CH, PhSi), 127.7 (4CH, PhSi), 125.1 (CH, =*C*H), 124.5 (2CH, =*C*H), 124.4 (CH, =*C*H), 64.3 (CH, $(CH_3)_2$C(O)*C*H), 63.7 (CH_2, *C*H_2OSi), 58.4 (C, $(CH_3)_2$*C*(O)CH), 39.9 (CH_2), 39.8 (CH_2), 36.4 (CH_2), 38.4 (CH_2), 35.9 (CH_2), 31.1 (CH_2), 28.4 ($2CH_2$), 27.6 (CH_2), 27.0 ($3CH_3$, (*C*$H_3)_3$CSi), 26.8 (CH_2), 25.0 (2 CH_3, (*C*$H_3)_2$*C*(O)), 19.4 (C, $(CH_3)_3$*C*Si), 18.9 (CH_3), 16.2 ($2CH_3$), 16.0 (CH_3); HRMS (ESI+) : Calcd for $C_{43}H_{65}O_2Si$, 641.4748, found 641.4733.

(4*E*,8*E*,12*E,*16*E*)-20-[(*tert*-Butyldiphenylsilyl)oxy]-4,8,13,17-tetramethylicosa-4,8,12,16-tetraenal (26): A solution of epoxide **25** (1.60 g, 2.5 mmol) in diethyl ether (20 mL) was added dropwise to a suspension of periodic acid (0.65 g, 2.85 mmol) in diethyl ether (10 mL). The reaction mixture was stirred 2 h at 20 °C. Aqueous $NaHCO_3$ (30 mL) was added and the mixture was extracted with AcOEt (3 × 50 mL). The combined organic layers were washed with brine (10 mL), dried over $MgSO_4$ and concentrated under reduced pressure. The crude product was purified by chromatography over silica gel eluting with petroleum ether and then petroleum ether/Et_2O 95:5 to give the aldehyde **26** as a colorless oil (953 mg, 64%). ^{1}H NMR ($CDCl_3$, 300 MHz) δ 9.75 (t, *J* = 1.8 Hz, 1H, C*H*O), 7.67 (dd, *J* = 7.5, 1.7 Hz, 4H, SiPh), 7.50–7.30 (m, 6H, SiPh), 5.20–5.06 (m, 4H, =C*H*), 3.64 (t, *J* = 6.4 Hz, 2H, C*H*$_2$OSi), 2.50 (t, *J* = 7.1 Hz, 2H, C*H*$_2$CHO), 2.31 (t, *J* = 7.3 Hz, 2H, C*H*$_2$CH$_2$CHO), 2.15-1.85 (m, 14H, =HCC*H*$_2$C*H*$_2$C(CH_3)), 1.77–1.55 (m, 14H, C*H*$_2$CH$_2$OSi, =CH(C*H*$_3$)), 1.05 (s, 9H, (C*H*$_3)_3$CSi); ^{13}C NMR (75 MHz, $CDCl_3$) δ 202.7 (C, *C*HO), 135.7 (4CH, SiPh), 135.3 (2C, CSi), 134.9 (C, =*C*(CH_3)), 134.7 (C, =*C*(CH_3)), 134.3 (C, =*C*(CH_3)), 133.0 (C, =*C*(CH_3)), 129.6 (2CH, SiPh), 127.7 (4CH, SiPh), 125.6 (CH, =*C*H), 124.7 (CH, =*C*H), 124.5 (CH, =*C*H), 124.4 (CH, =*C*H), 63.7 (CH_2, *C*H_2OSi), 42.3 (CH_2), 39.9 (CH_2), 39.7 (CH_2), 35.9 (CH_2), 32.0 (CH_2), 28.4 ($2CH_2$), 27.2 ($3CH_3$, (*C*$H_3)_3$CSi), 26.8 (CH_2), 26.7 (CH_2), 19.4 (C, $(CH_3)_3$*C*Si) 16.2 ($2CH_3$), 16.1 (CH_3), 16.0 (CH_3); IR (film, cm^{-1}) ν : 3071, 2960, 2930, 2856, 1726, 1666, 1589, 1472,

1446, 1427, 1388, 1360, 1187, 1112, 1105, 1089, 1064, 1029, 1007, 998, 939, 823, 741, 709 ; HRMS (ESI+) : Calcd for $C_{40}H_{58}O_2SiNa$, 621.4098, found 621.4080.

(6*E*,10*E*,14*E*,18*E*)-19-{3-[(*tert*-Butyldiphenylsilyl)oxy]propyl}-2-($^2H_3$)methyl-6,10,15-trimethyl (1,1-$^2H_2$)icosa-1,6,10,14,18-pentaen-3-ol (27). A solution of *n*-butyllithium (1.3 mL, 2.5 M, 2.78 mmol) was added dropwise to a solution of trisylhydrazone **14** (435 mg, 1.26 mmol) in DME (4 mL) and TMEDA (0.65 mL, 4.4 mmol) cooled at −78 °C. The reaction mixture was stirred at −78 °C for 30 min. and warmed up to 0 °C. After few min the yellow reaction mixture turned green and a strong gas evolution took place. After completion of the nitrogen gas evolution, the flask was replaced in the –78 °C cool bath. A solution of aldehyde **26** (590 mg, 0.96 mmol) in DME (1 mL) was added dropwise. The mixture was stirred at −78 °C for 1h and the temperature was then raised up to 20 °C and the reaction mixture was quenched by a solution of 0.1 N HCl (2 mL). The mixture was taken up into Et_2O (30 mL), the phases were separated and the aqueous layer extracted with Et_2O (3 × 50 mL). The combined organic layers were washed with saturated $NaHCO_3$ (5 mL), brine (5 mL), dried over $MgSO_4$ and concentrated under reduced pressure. The crude product was purified by chromatography over silica gel eluting sequentially with petroleum ether, petroleum ether/Et_2O 95:5 and finally petroleum ether/Et_2O 90:10 to provide alcohol **27** (305 mg, 63%) as a colorless oil. ^1H NMR ($CDCl_3$, 400 MHz) δ 7.71-7.65 (m, 4H, SiPh), 7.46-7.33 (m, 6H, SiPh), 5.23-5.08 (m, 4H, =C*H*), 4.05 (t, *J* = 6.0 Hz, 1H, C*H*OH), 3.66 (t, *J* = 6.5 Hz, 2H, C*H*$_2$OSi), 2.18-1.84 (m, 16 H, =CC*H*$_2$C*H*$_2$C(CH_3)), 1.77-1.49 (m, 16 H, C*H*$_2$CH$_2$OSi, =CH(C*H*$_3$), C*H*$_2$CH(OH)), 1.07 (s, 9H, (C*H*$_3$)$_3$CSi); ^{13}C NMR ($CDCl_3$, 100 MHz) δ 147.4 (C, D$_2$C=*C(*CD$_3$*)*), 135.7 (4CH), 135.2 (2C, CSi), 135.1 (C, =*C*(CH_3)), 134.8 (C, =*C*(CH_3)), 134.7 (C, =*C*(CH_3)), 134.3(C, =*C*(CH_3)), 129.6 (2CH, SiPh), 127.7 (4CH, SiPh), 124.9 (CH, =*C*H), 124.5 (2CH, =*C*H), 124.4 (CH, =*C*H), 75.7 (CH, *C*HOH), 63.7 (CH_2, *C*H$_2$OSi), 39.9 (CH_2), 39.8 (CH_2), 35.9 (CH_2), 35.8 (CH_2), 33.3 (CH_2), 31.1 (CH_2), 28.4 (2CH_2), 27.0 (3CH_3, (*C*H$_3$)$_3$CSi), 26.8 (CH_2), 26.7 (CH_2), 19.3 (C, (CH_3)$_3$*C*Si), 16.2 (CH_3), 16.2 (2CH_3),16.1 (CH_3); IR (film, cm^{-1}) ν : 3600-3200, 3072, 2961, 2929, 2856, 1667, 1590, 1487, 1473, 1462, 1449, 1428, 1390, 1361, 1330, 1305, 1258, 1188, 1112, 1105, 1090, 1064, 1029, 1007, 998, 940, 823, 741, 709 ; HRMS (ESI+) : Calcd for $C_{43}H_{59}D_5O_2SiNa$, 668.4881, found 668.4846.

(4*E*,8*E*,12*E*,16*E*)-21-($^2H_3$)Methyl-4,8,13,17-tetramethyl(22,22,22-$^2H_3$)docosa-4,8,12,16,20-pentaen-1-ol (**28**). Thionyl chloride (250 mg, 2.1 mmol, 14 equiv) was added to a solution cooled at 0 °C of alcohol **27**, (101 mg, 0.15 mmol) in diethyl ether (10 mL). The mixture was stirred at 0 °C for 4 h and then quenched by a solution of $NaHCO_3$ (2 mL). The mixture was extracted with Et_2O (3 X 15 mL), dried over $MgSO_4$ and concentrated under reduced pressure to give a yellow oil (100 mg). The crude product was taken in dried THF (2 mL) and added to a suspension of $LiAlD_4$ (65 mg, 1.5 mmol) in THF (1 mL). The mixture was heated at reflux for16 h and cooled to 0 °C. Few drops of ethyl acetate were added to destroy the excess of hydride and THF (10 mL) was added. A saturated aqueous solution of sodium sulfate was then added with efficient stirring until most of the salts precipitated. Filtration and concentration under reduced pressure gave an oil which was chromatographed over silica gel eluting with petroleum ether/Et_2O 95:5 to provide alcohol as a colorless oil (24 mg, 41%). ^1H NMR ($CDCl_3$, 400 MHz) δ 5.23-5.08 (m, 5H, =C*H*), 3.62 (t, *J* = 6.4 Hz, 2H, C*H*$_2$OH), 2.15-1.80 (m, 18H, =CC*H*$_2$C*H*$_2$C(CH_3)), 1.70-1.60 (m, 14H, (C*H*$_3$)C=, C*H*$_2$CH$_2$OH); ^{13}C NMR (101 MHz, $CDCl_3$) δ 135.3 (C, =*C*(CH_3)), 134.9 (2C, =*C*(CH_3)), 134.7 (C, =*C*(CH_3)), 131.2 (C, (CD_3)$_2$*C*=), 124.9 (CH, =*C*H), 124.6 (2CH,

=*C*H), 124.4 (2CH, =*C*H), 62.9 (CH_2, *C*H_2OH), 39.9 ($2CH_2$), 39.8 (CH_2), 36.1 (CH_2), 30.9 (CH_2), 28.4 ($2CH_2$), 26.9 (CH_2), 26.8 (CH_2), 26.7(CH_2), 16.1 ($3CH_3$), 16.0 (CH_3); IR (film, cm^{-1}) ν : 3500-3100, 3053, 2980, 2960, 2935, 2918, 2912, 2856, 2843, 2225, 2190, 2140, 2062, 1666, 1558, 1454, 1450, 1439, 1432, 1383, 1360, 1331, 1260, 1157, 1149, 1108, 1090, 1061, 1048, 1020, 916, 862, 846, 841; Calcd for $C_{27}H_{40}D_6ONa$, 415.3817, found 415.3797.

^{1}H NMR Spectrum of compound **1**

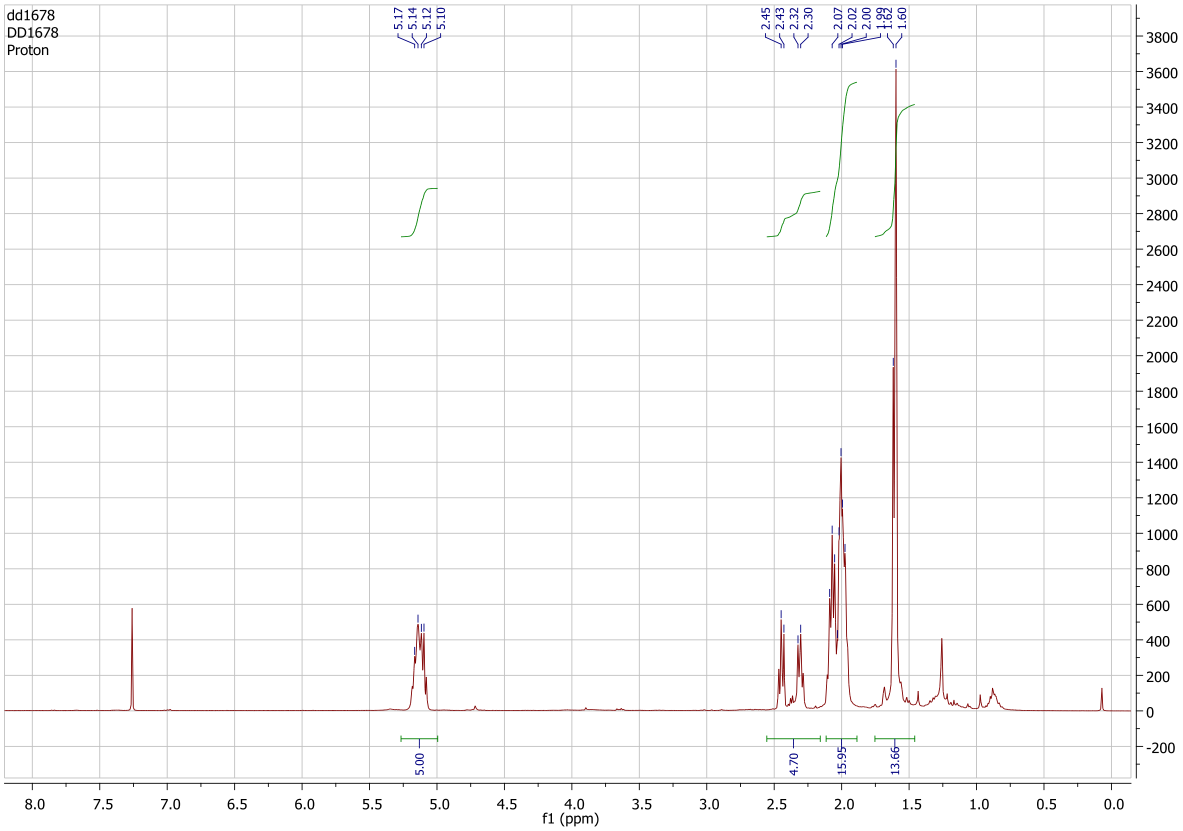

^{13}C NMR Spectrum of compound **1.**

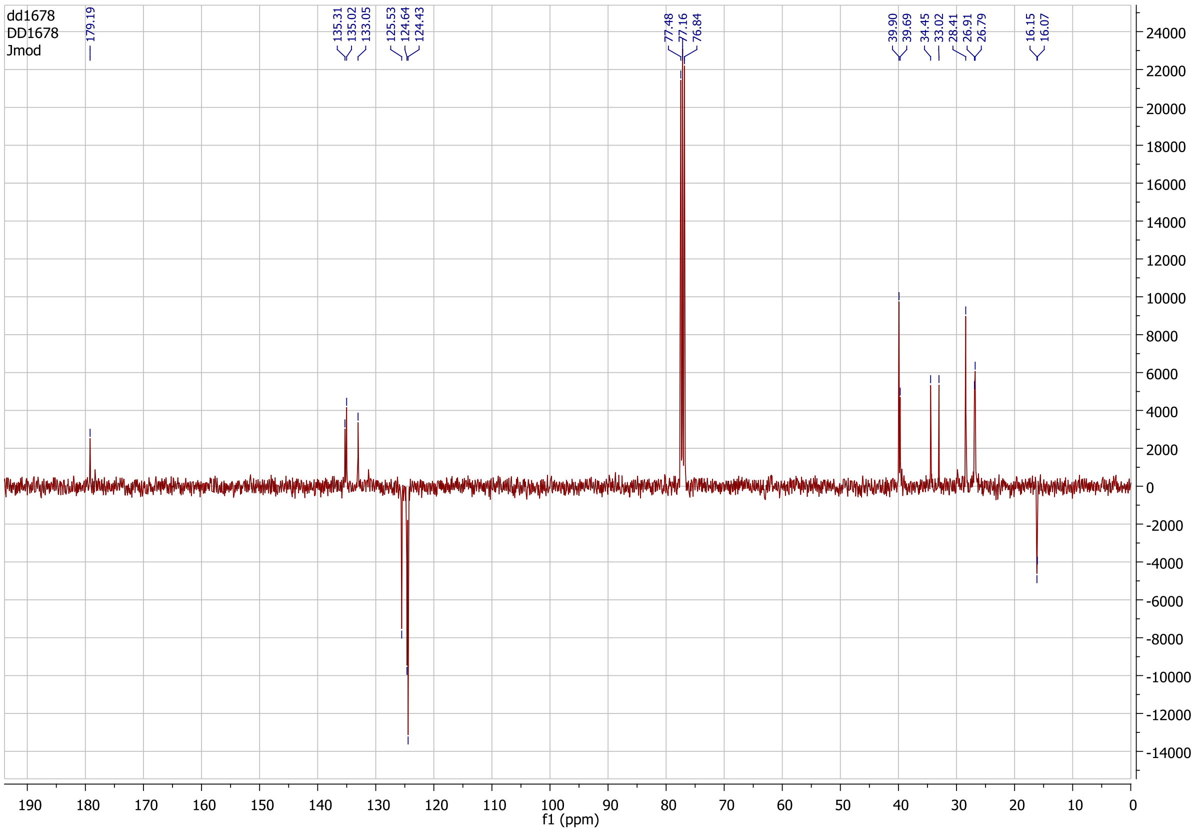

^{1}H NMR Spectrum of compound **3**.

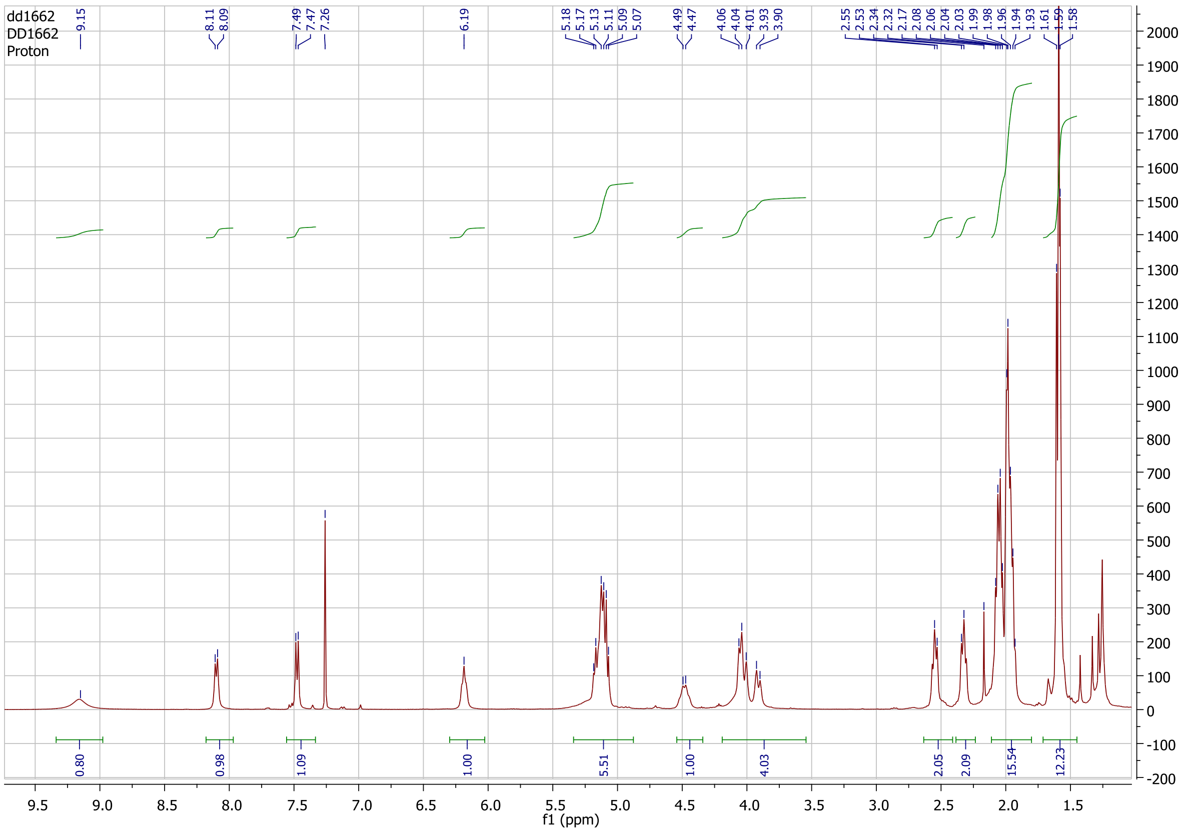

^{13}C NMR Spectrum of compound **3**.

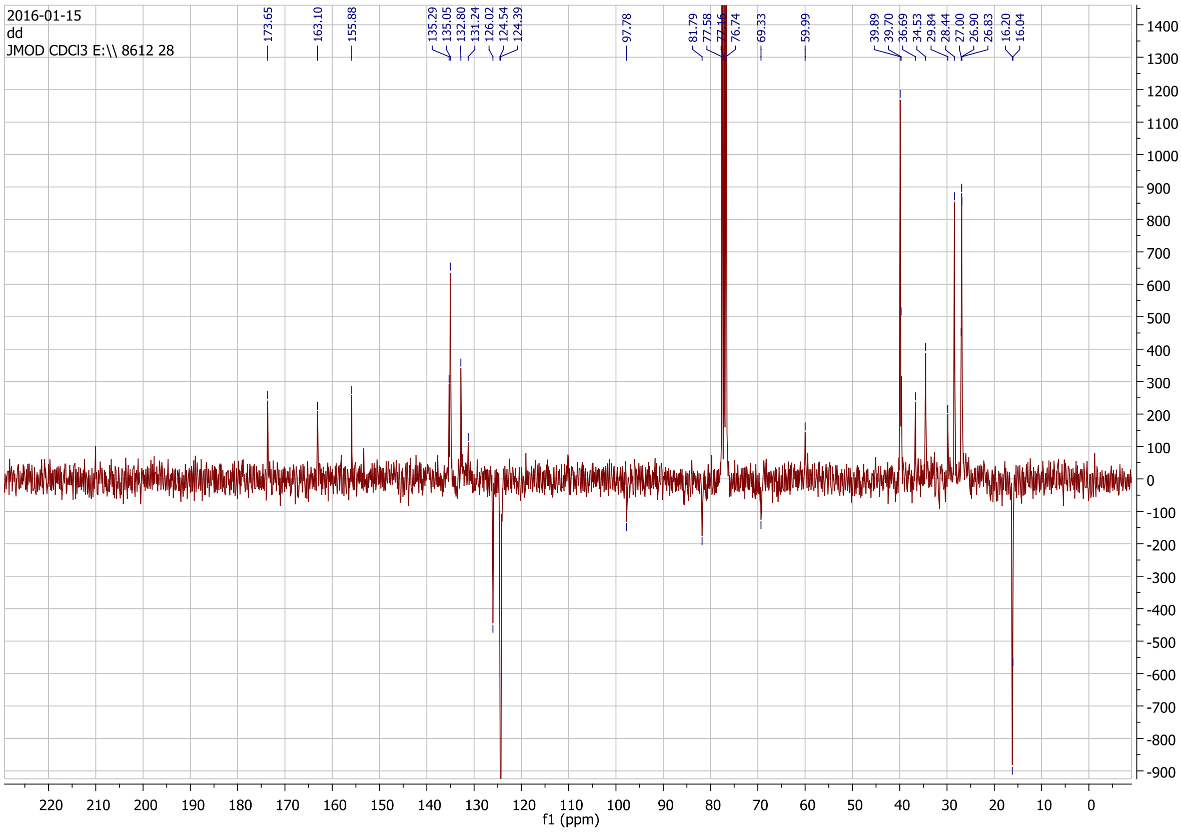

^{1}H NMR Spectrum of compound **11**.

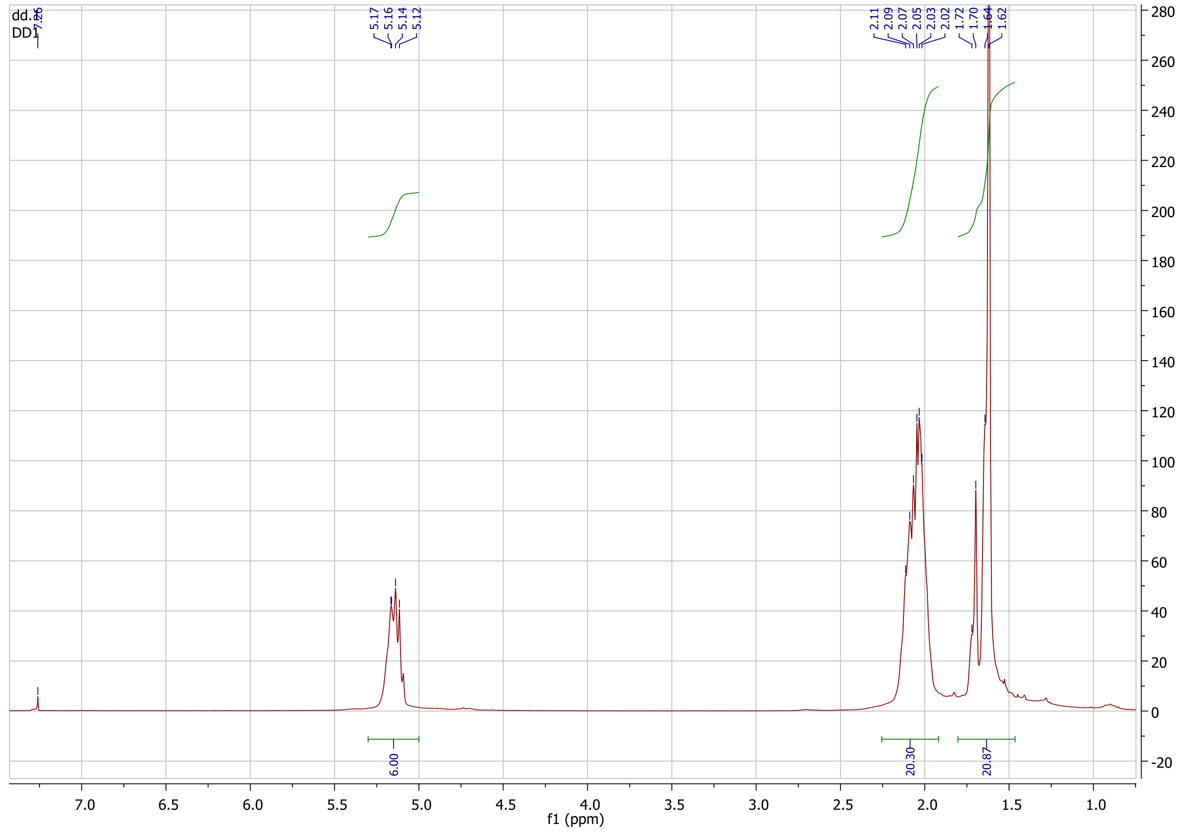

^{13}C NMR Spectrum of compound **11**.

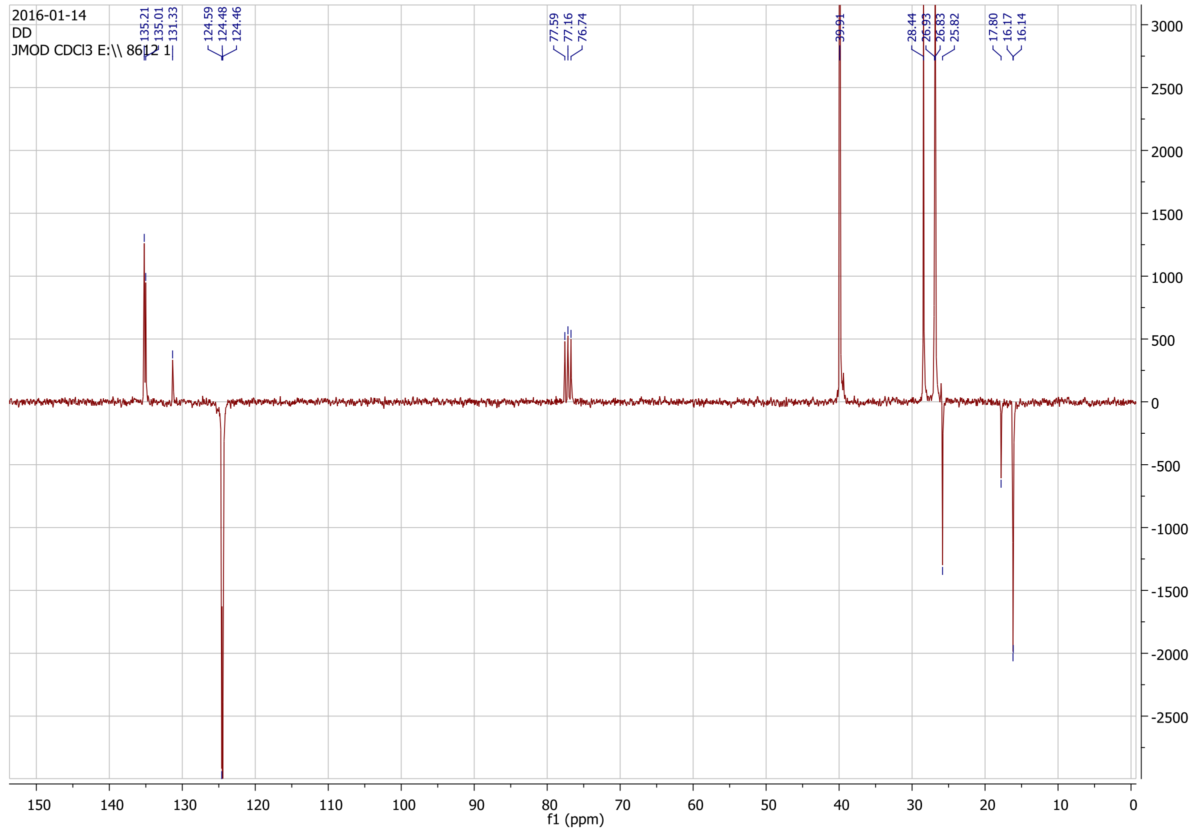

^{1}H NMR Spectrum of compound **25**.

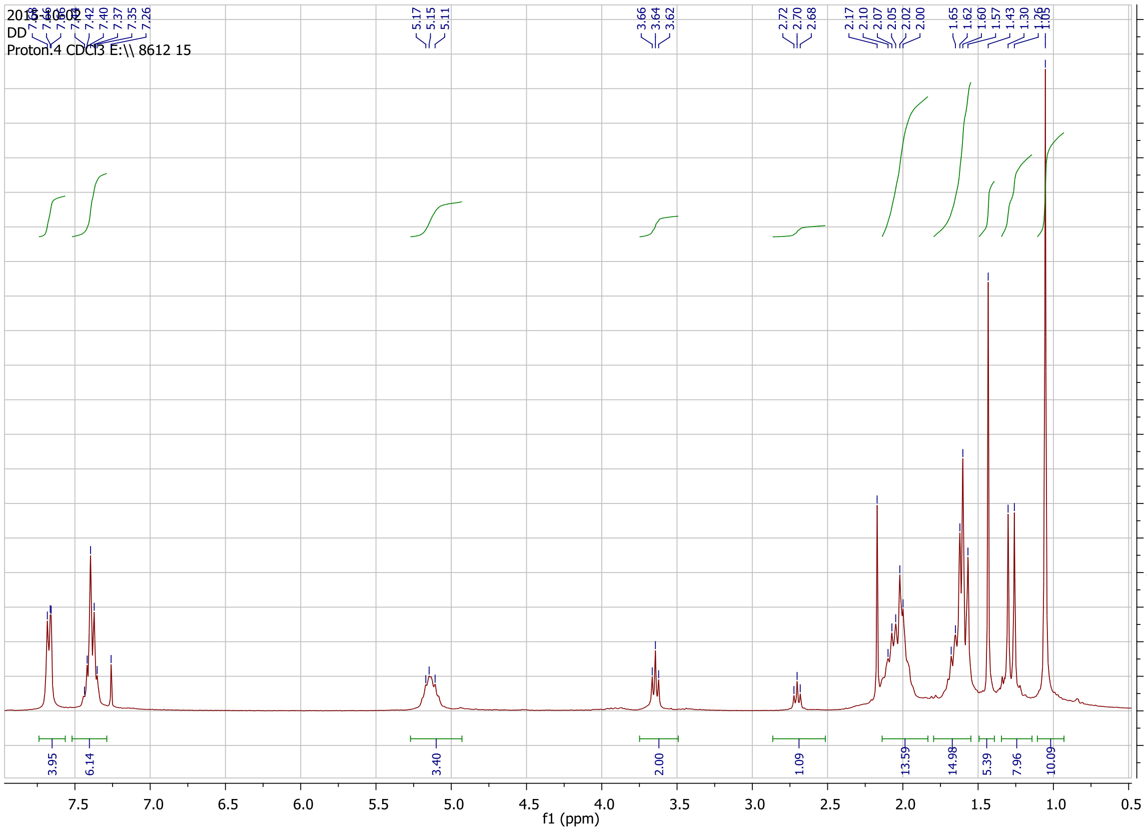

^{13}C NMR Spectrum of compound **25**.

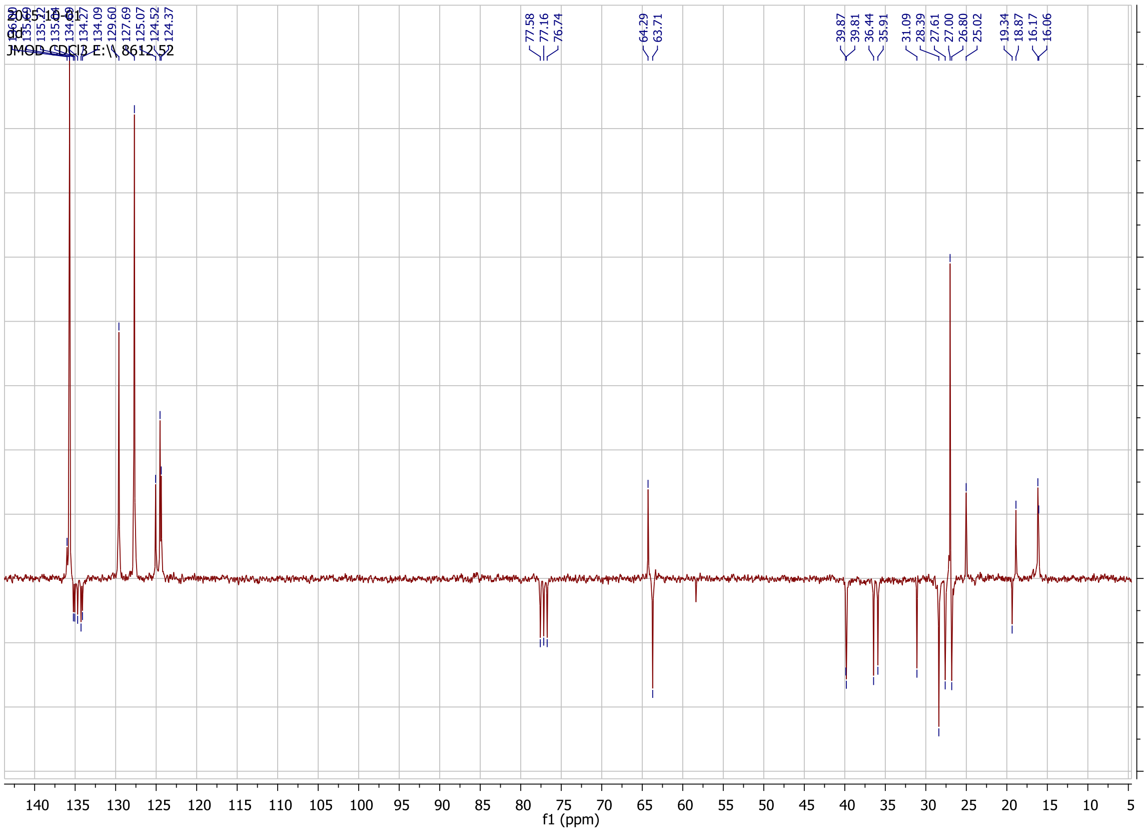

^{1}H NMR Spectrum of compound **27**.

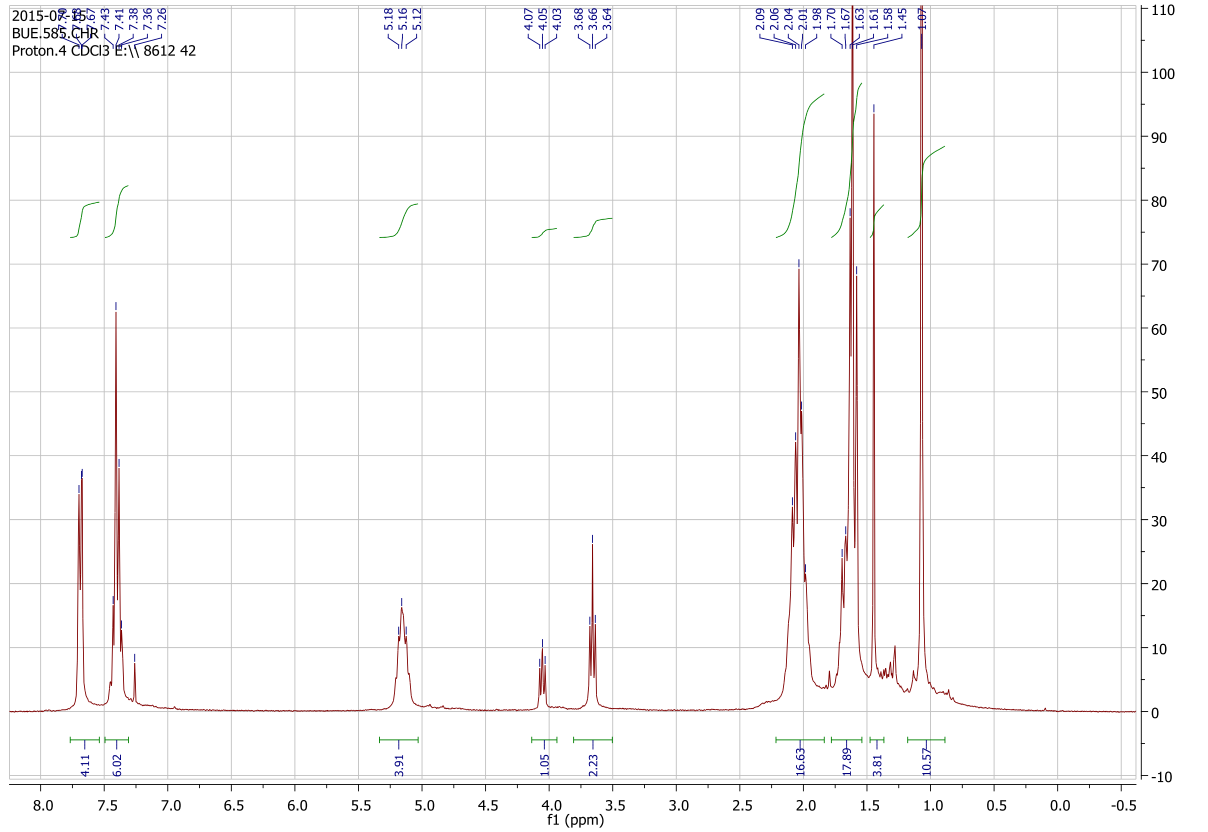

^{13}C NMR Spectrum of compound **27**.

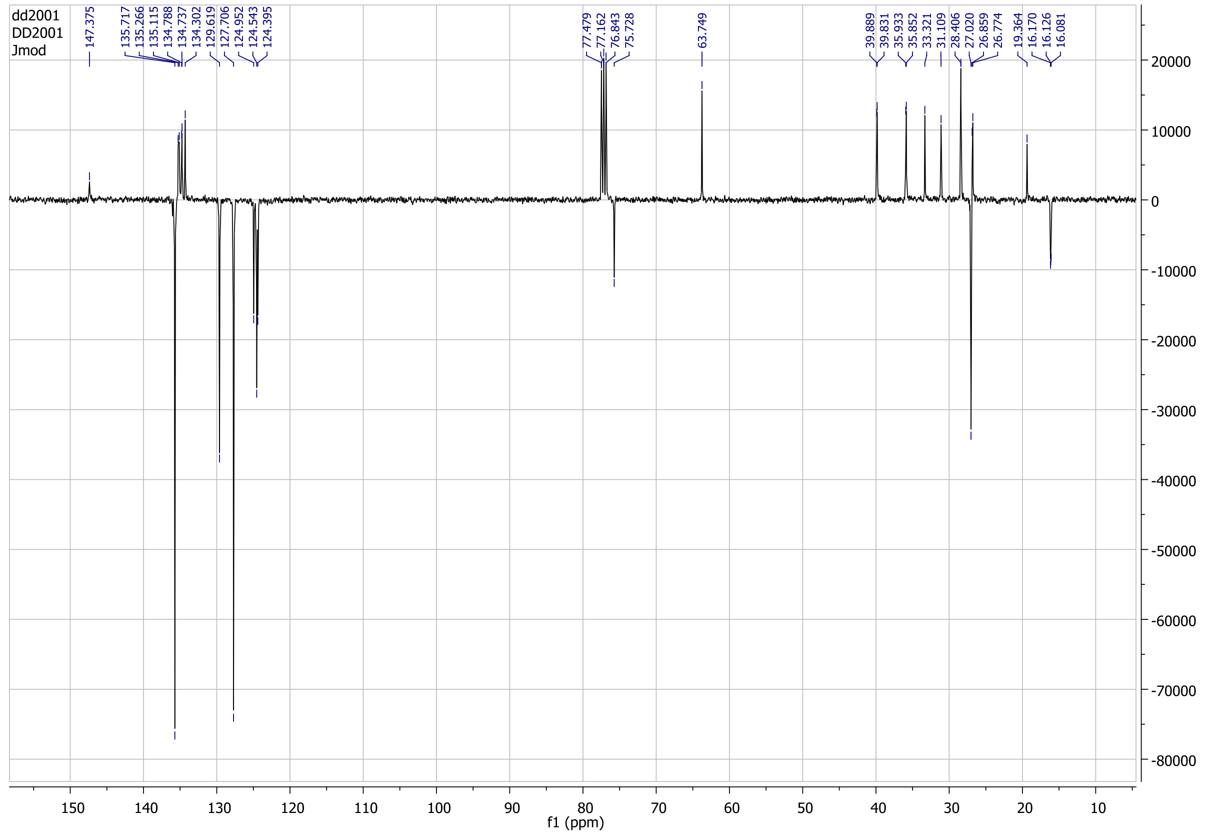

^{1}H NMR Spectrum of compound **28**.

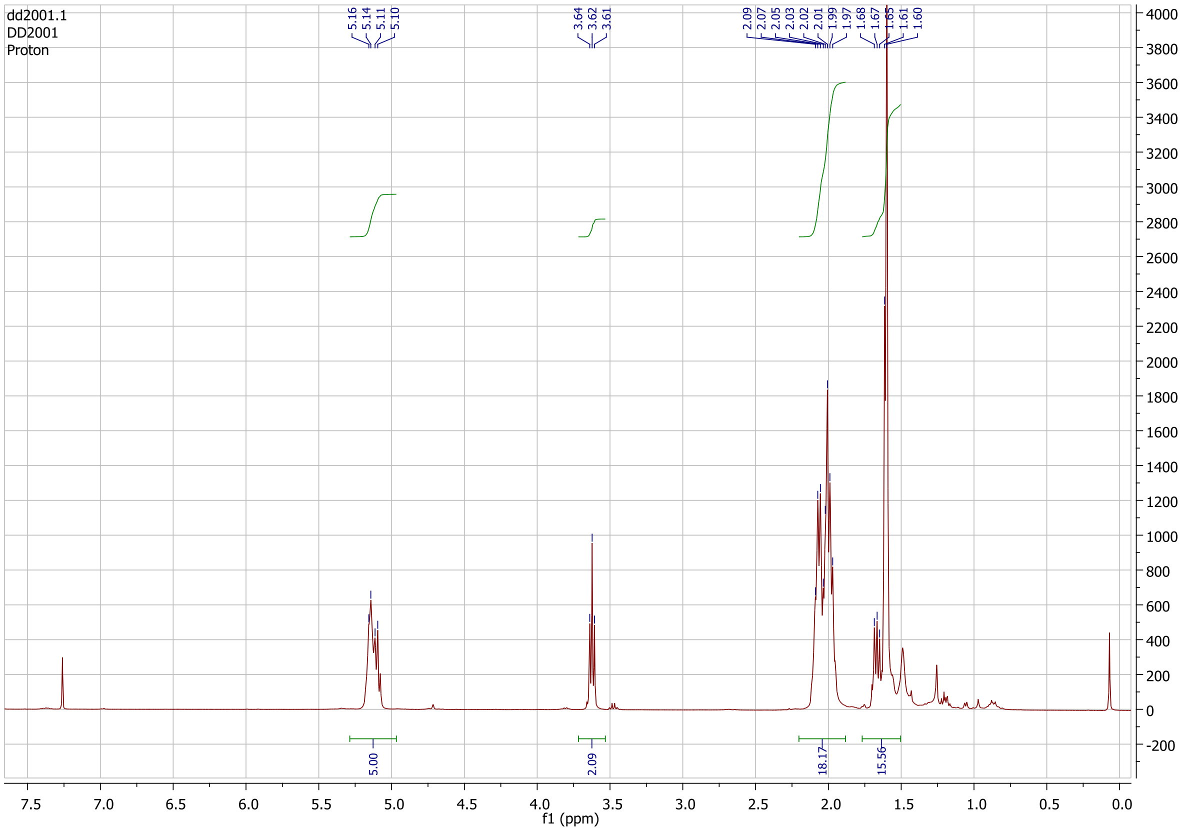

^{13}C NMR Spectrum of compound **28**.

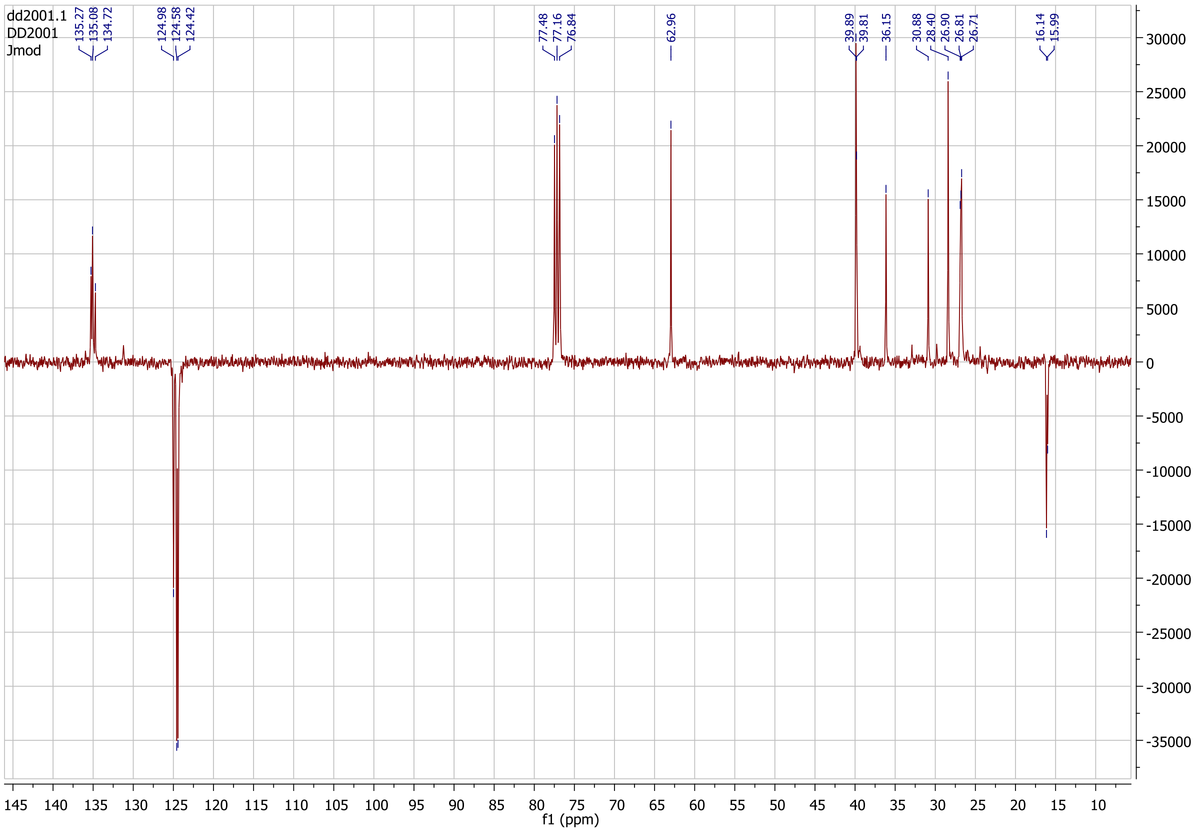

Reference

[1] . Sen, S. E.; Prestwich, G. D. *J. Am. Chem. Soc*. **1989**, *111*, 1508-1510. doi: 10.1021/ja00186a062
